# A decision support framework for reliable early prediction of long-term cumulative milk yield in dairy cows

**DOI:** 10.5713/ab.260424

**Published:** 2026-07-27

**Authors:** Jae-Woo Song, Dong-Hyeon Kim, Seung-Hyun Lee, Seongwon Seo, Wang-Hee Lee

**Affiliations:** 1 Department of Smart Agriculture Systems Machinery Engineering, Chungnam National University, Daejeon, Korea; 2 Department of Smart Agriculture Systems, Chungnam National University, Daejeon, Korea; 3 Division of Animal and Dairy Sciences, Chungnam National University, Daejeon, Korea

**Keywords:** Long-term Cumulative Milk Yield, Machine Learning Algorithms, Milk Yield Prediction, Parity-specific Modeling, Smart Dairy Farming, Statistical Model

## Abstract

**Objective:**

Early prediction of cumulative milk yield from initial lactation records enables timely culling, selective breeding, and optimized feed allocation, thereby improving herd efficiency and farm profitability. However, predicting long-term production remains challenging because of high variability in early-lactation patterns and limited available data. This study aimed to develop and compare predictive models for estimating long-term cumulative milk yield from daily production records and to identify the optimal approach and minimum days in milk (DIM) threshold for reliable prediction.

**Methods:**

We used 211 lactation datasets with complete records up to 305 DIM, selected from daily milk yield records of 727 dairy cows. Six models were evaluated: three statistical methods (simple averaging, adjustment factor, and correction coefficient) and three ensemble machine learning algorithms (CatBoost, XGBoost, and Random Forest). Because lactation patterns differ between primiparous and multiparous cows, parity-specific models were also developed. Predictive performance was assessed using mean absolute percentage error (MAPE) between predicted and actual yields.

**Results:**

The 305-day cumulative milk yield was predicted within an 11% error margin across all base DIMs, with the smallest discrepancy of 0.5 kg observed in the XGBoost model. Machine learning models showed better performance during early lactation (MAPE 8.878 at base DIM 30 for parity ≥2), whereas statistical models achieved higher accuracy after base DIM 120 (MAPE 4.311 at base DIM 150 for parity 1).

**Conclusion:**

This study provides a lactation stage-dependent and parity-specific prediction framework that bridges early-lactation uncertainty and long-term productivity estimation. By enabling model selection according to data availability and lactation stage, the proposed approach supports the practical application of predictive analytics in dairy farming and provides a decision-support framework for early culling, feeding optimization, and productivity-based herd management.

## INTRODUCTION

In the dairy industry, the individual milk yield is a key indicator of farm productivity and economic performance, and the cumulative milk yield over the first 305 days after calving is widely used as a standard metric to evaluate the production capacity of dairy cows [1,2]. Therefore, early prediction of lactation milk yield offers critical decision support for dairy herd management, including selective breeding, nutritional optimization, reproductive scheduling, and culling strategies [3]. Specifically, predicting lifetime productivity from early-lactation records enables timely identification of underperforming individuals, facilitates proactive resource allocation, and enhances overall herd efficiency [4]. Recent advances in automated milking systems and sensor-based dairy technologies have enabled large time-series datasets for individual-level milk yield [5], creating new opportunities for predictive modeling of dairy productivity [6]. This technological shift necessitates advanced predictive methods that enable early estimation of long-term performance beyond conventional retrospective analysis.

Accurately predicting total milk yield from early-lactation records is inherently challenging due to high inter-individual variability in milk production traits and limited information on overall lactation patterns, including peak yield and the rate of post-peak decline. Traditional approaches rely primarily on parametric lactation curve models that describe temporal production patterns using predefined regression-based functions [7–9]. While these models offer the advantage of biological interpretability [10], they typically assume a fixed functional form derived from population-level data and apply it uniformly across individuals, which reduces robustness across individual measures. Consequently, their ability to capture individual-specific deviations is limited, particularly during early lactation when milk yield trajectories are more variable and less predictable [9]. To address these limitations, either individually adjusted statistical estimations or machine learning (ML) based approaches can be employed [11].

Beyond traditional lactation curve models, a range of statistical and ML approaches have been explored to improve milk yield prediction. Statistical methods offer advantages, such as interpretability, computational efficiency, and stable performance under limited data conditions, making them suitable for practical farm applications [3,12,13]. ML algorithms, such as Random Forest, gradient boosting, XGBoost, and artificial neural networks, have demonstrated strong potential for capturing complex nonlinear relationships and individual variability in biological time-series data [14–16]. Although both methods are useful, they have distinct weaknesses: statistical models often miss complex nonlinear patterns and individual quirks early, whereas ML requires vast amounts of data to be reliable. As their effectiveness shifts based on the lactation stage and data volume, a systematic comparison is necessary to determine which performs better in specific scenarios. Although conventional lactation curve models have been widely used for prediction from partial records, less attention has been given to how the relative performance of statistical and ML approaches changes according to the amount of early- to mid-lactation information available. Unlike parametric curve models that estimate a predefined functional trajectory, the framework evaluated in this study directly predicts cumulative milk yield using summary variables derived from the observed records. By evaluating multiple base days in milk (DIM) values and parity groups, this approach provides a practical basis for selecting an appropriate prediction method according to the amount of lactation information available. Furthermore, the effect of parity (the difference in yield patterns between first-time and experienced cows) remains a critically underexplored variable. Milk yield patterns differ by parity: primiparous cows show a slower rise to a delayed peak, whereas multiparous cows reach higher early yields but experience a faster post-peak decline [17,18]. These physiological variations create distinct lactation curves and persistence levels across the two groups [19]. While some studies have used parity-specific strategies, they are often inconsistent, and there is currently no unified framework that systematically compares prediction performance with and without explicit parity differentiation [3,14]. These gaps hinder the accurate long-term yield estimates for individual cows. Although modern data and modeling techniques allow for parity-specific analysis, we still lack a comprehensive evaluation of how statistical and ML models perform under these conditions, suggesting that a systematic comparison is needed to account for parity-dependent variations in production.

This study aimed to develop and validate predictive models for estimating long-term cumulative milk yield using daily early- to mid-lactation production records. We compared six modeling approaches: three statistical methods—(i) simple moving average, (ii) adjustment factors derived from population-level lactation curves, and (iii) empirical correction coefficients based on observed-to-predicted ratios—and three ensemble ML algorithms (CatBoost, XGBoost, Random Forest). Model performance was evaluated across incremental DIM thresholds to identify the earliest reliable prediction window for lifetime productivity forecasting. These findings will enable early-lactation management decisions through the comparative evaluation of statistical and ML approaches that provide scalable and reliable yield prediction tools adaptable to diverse farm capacities.

## MATERIALS AND METHODS

### Data acquisition and screening

Daily milk production data from dairy cows measured over a three-year period from January 2016 to December 31, 2018, were obtained from four commercial farms located in Boryeong and Cheonan, Chungcheongnam-do, Wonju, Gangwon-do, Gimcheon, Gyeongsangbuk-do, and Cheonan, Chungcheongnam-do, South Korea. Among the four farms, only the farm located in Boryeong used a conventional milking parlor system (DeLaval International), whereas the remaining farms used a robotic milking system (Lely Industries). Daily milk production from 727 dairy cows was digitized and stored using radio frequency identification (RFID) tags. A total of 307,053 daily milk production time-series data points, with an average parity of 2.46, were obtained. Because individual cows could contribute records from different parities during the study period, each unique combination of cow identification number and parity was treated as a separate lactation dataset. The records from 727 cows therefore comprised 1,346 lactation datasets. For model development, only datasets containing valid daily milk yield records continuously from DIM 1 to 305 were retained. Days without recorded milk yield values and records with a milk yield of zero were treated as missing observations because zero values were considered implausible and likely attributable to recording or sensor errors. A total of 1,117 datasets were excluded because they lacked one or more valid observations within DIM 1–305. Many of these datasets had been recorded only after lactation was already in progress and therefore lacked early-lactation records. This screening process resulted in 229 complete lactation datasets. Of these, 18 datasets with parity 5 or higher were excluded to maintain adequate sample sizes within the parity groups. The final dataset therefore comprised 211 complete lactation records, including 71, 69, 47, and 24 datasets for parities 1, 2, 3, and 4, respectively.

### Model development

#### Daily average-based prediction: simple model

Because the main objective of this study was to predict total cumulative milk production over 305 days of lactation, we developed a simple model (SM) based primarily on the average daily milk yield during this period. Specifically, this approach calculates the average daily flow by dividing the cumulative flow for 150 days at 30-day intervals by DIM and then multiplying by 305 to predict the cumulative flow for 305 days (Eq. 1).


(1) $$\text{Predicted total milk yield}=\left(\frac{\text{Cumulative milk }{\text{yield}}_{i}}{\text{Days in }{\text{milk}}_{i}}\right)\times 305$$

where *i* is the number of days in milk used as the basis for prediction (*i* = 30, 60, 90, 120, and 150).

#### Modified existing method-based prediction: adjustment factor-based model

We implemented an adjustment factor-based model (AFM) to predict total milk yield over 305 days by applying adjustment factors reported in a previous study [20], which were derived from 4,264,347 milk yield records collected between 2000 and 2005. In that study, adjustment factors for the calving and lactation stages were developed based on the least-squares mean calculated using a general linear model for cases where parity was one and two or more. However, because the milking data used in this study did not contain information other than parity, the total milk yield was predicted using the average value of the adjustment factor from a previous study (Supplement 1). To predict the entire parity, the average value of the adjustment factor for parity was one, two, or more. Because information on calving season and exact age in months was unavailable, the adjustment factors could not be applied using the full stratification scheme described in the original study. Therefore, the AFM was implemented using averaged adjustment factors, and its results represent the performance of this approach under the limited information available in the present dataset.

#### Correction of a simple method-based prediction: correction coefficient-based model

To improve the prediction performance, correction coefficients for each DIM were estimated based on the results predicted by the SM. The average of 10 correction coefficients estimated based on 10-fold cross-validation was used as the final correction coefficient to evaluate model performance (Figure 1). The correction coefficients were estimated by dividing the SM results of the training data by the actual total milk yield up to 305 DIM (Eq. 2). The correction coefficient-based model (CCM) predicts total milk yield by dividing the estimated correction coefficient by the SM results of the dairy cattle being predicted (Eq. 3).


(2) $$\text{Correction coefficient}=\left(\frac{\text{Simple model result}}{\text{Total milk yield}}\right)$$

(3) $$\text{Predicted total milk yield}=\left(\frac{\text{Simple model result}}{\text{Correction coefficient}}\right)$$

#### Machine learning algorithm-based prediction: machine learning-based model

The final approach implemented a ML-based model using three ML algorithms (CatBoost, XGBoost, and Random Forest) to predict the long-term milk yield. CatBoost is a gradient boosting method optimized for handling categorical variables with minimal preprocessing, whereas XGBoost is an efficient boosting algorithm that integrates regularization and parallel computation to enhance accuracy and prevent overfitting [21,22]. Random Forest is an ensemble of decision trees that improves prediction stability by reducing variance through aggregated learning [23]. Because each algorithm requires distinct hyperparameter configurations, we performed a grid search using the GridSearchCV function from the scikit-learn Python library to identify the optimal parameter combinations for each base DIM [24] (Supplement 2). Hyperparameters were optimized using 10-fold cross-validation, and the best combination was selected based on the lowest prediction error. For each algorithm, the predictor variables were derived from daily milk yield data. These included the initial milk yield (defined as the average yield from days 5 to 30), cumulative milk yield over a 10-day period, and the slope of the linear regression for milk yield over the same duration.

### Model application

To assess how the prediction performance varies with DIM and to determine the optimal prediction time for a total 305-day milk yield, the cumulative milk yield was evaluated from 200 to 300 DIM at 10-day intervals. The SM, CCM, and ML-based models were applied across these prediction horizons; however, the AFM could not be used in this analysis because only an adjustment factor based on 305 days existed [20]. To address parity-dependent variations in lactation patterns, models were developed for all parities combined and for parity-specific groups. Before defining the parity groups, we examined the ratio of the cumulative milk yield predicted by the SM to the actual cumulative milk yield for each parity and base DIM. Parity 1 showed a distinct pattern from parities 2–4, whereas parities 2, 3, and 4 showed more comparable ratio ranges and trends across the evaluated base DIMs (Supplement 3). Therefore, parity 1 was treated as a separate primiparous group (n = 71), while parities 2–4 were combined as a multiparous group (n = 140) because the individual sample sizes, particularly for parity 4, were insufficient for stable model development. Owing to the limitations of the available data size, 10-fold cross-validation was performed when estimating the correction coefficient of the CCM and developing the ML models.

### Model performance evaluation

Mean absolute percentage error (MAPE) was used to evaluate the performance of milk yield prediction models. Calculated as the average of the absolute difference between the actual and predicted values and expressed as a percentage, MAPE is a standard metric for dairy yield prediction; lower values indicate superior model performance [25,26]. In addition, to compare the predicted and measured values for total milk production during the given period for population size, Tukey’s honestly significant difference post-hoc test was performed with the assumption of normality, which was satisfied by the number of sample size [27]. When the sample sizes between the groups being compared were unequal, we used the Games–Howell post-hoc test [28]. Statistical analysis was performed using Python 3.9.13, and the significance level was set at p<0.05.

## RESULTS

### Predicting the total milk production at 305 days

Across all the statistical ML models, the prediction accuracy for total milk yield improved as the base DIM increased, with the lowest MAPE consistently observed at 150 DIM (Table 1). Among the statistical models, the CCM exhibited the highest accuracy, with the MAPE declining from 13.82% to 6.30% as the DIM increased. In contrast, SM showed the highest error rates. ML models followed a similar trajectory: CatBoost generally outperformed the other ML methods, achieving a minimum MAPE of 5.28% at 150 DIM, whereas RF and XGBoost showed slightly lower overall performances. Although the mean MAPE generally decreased as the base DIM increased, the maximum individual-cow APE increased slightly at 120 DIM compared with 90 DIM for several statistical and ML models. However, this pattern was not consistently observed across all algorithms and appeared to reflect individual-level variation rather than an overall decline in mean predictive performance.

Model performance varied by parity, particularly within statistical models (Table 2). For Parity 1, the accuracy improved sharply as DIM increased; however, for Parities 2–4 at 150 DIM, the MAPE of SM (10.81%) was approximately double that of the CCM and AFM. The CCM remained the most robust statistical model across all parities, with the MAPE staying within 0.16% of the best-performing models in specific categories. In the ML analysis, CatBoost maintained a superior performance for Parity 1, although XGBoost and RF showed localized advantages at 60 and 90 DIM for Parities 2–4. Notably, the minimum MAPE for Parity 1 was the highest in the SM at 30 DIM (2.52), whereas all ML models maintained a lower minimum MAPE (<0.5) across all parity groups.

Statistical significance testing revealed that the CCM and all ML models produced predicted values with no significant differences from the actual total milk yields at 305 days (Table 3). Conversely, SM and AFM tended to overestimate production by approximately 4%, with SM significantly underestimating the yield for Parity 1 at 30 DIM and overestimating it for Parities 2–4 (Table 4). While the ML models demonstrated high stability, with average predicted values closely mirroring actual yields regardless of DIM, the statistical models were more sensitive to the base DIM. Ultimately, the CCM at 150 DIM (Parity 1) and 30 DIM (Parities 2–4) provided the most accurate statistical estimations, whereas the ML models achieved optimal parity-specific proximity to actual averages at 90 DIM.

### Predicting the total milk production over days across the base days in milk

The SM and CCM were used to predict total milk yield from DIM 200 to 300 at 10-day intervals, whereas AFM could not be applied for this purpose as it was designed to predict total milk yield only at DIM 305. For the entire parity, the MAPE generally tended to increase as the prediction horizon lengthened across all base DIMs, with the exception of SM at base DIM 30 (Table 5). When comparing model performance, the CCM consistently exhibited a lower MAPE than the SM, except at base DIM 60, where both models showed similar performances. The highest accuracy was achieved when predicting the total milk yield over a 200-day period from a base DIM of 150, yielding a minimum MAPE of 2.547. In the ML analysis, all models followed the general trend of increasing MAPE with the extension of the prediction horizon. CatBoost demonstrated the highest predictive performance at earlier stages (base DIM: 30–90). However, at later stages (base DIM 120 and 150), XGBoost outperformed the other models in most scenarios, achieving its lowest MAPE (2.523) at base DIM 150 for the 200-day prediction.

The predictive accuracy for milk yield, evaluated using MAPE, was significantly influenced by the model algorithm, parity, and base DIM. For the statistical models, the MAPE generally exhibited an upward trend as the prediction horizon was extended, with the highest errors observed in the SM at base DIM 30 for both parity 1 (19.582) and Parity 2–4 (15.074) (Table 6). While CCM consistently outperformed SM in early lactation, its performance was comparable to that of the base DIM 90 onwards. In the ML framework, CatBoost was the most accurate for Parity 1, whereas RF exhibited the lowest performance at the base DIM of 30 (MAPE 10.834). Conversely, for Parities 2–4, the RF generally achieved a higher accuracy than the other ML models, although CatBoost remained superior in certain scenarios. Despite these algorithmic differences, both the statistical and ML models reached their peak performance at a base DIM of 150 for 200-day projections, where CCM (MAPE 1.810 for Parity 1; 2.397 for Parity 2–4) and XGBoost (MAPE 3.123 for Parity 2–4) emerged as the most precise predictors. Furthermore, the CC values estimated in the statistical models tended to decrease as the prediction period and base DIM decreased.

### Comparative analysis of statistical and machine learning models

A comparative analysis was conducted to evaluate the peak performances of the statistical and ML models across different parities and base DIMs. For the entire parity, ML models generally outperformed the statistical methods, although the MAPE gap narrowed from 4.469 to 1.026 as the base DIM increased (Figure 2). While both methodologies yielded predicted means that were closely aligned with the actual values, the ML models demonstrated higher stability with smaller standard deviations. However, a performance reversal was observed when analyzing specific parities; while ML models were superior at lower base DIMs (≤90), statistical methods achieved higher accuracy from base DIM 120 onwards for both Parity 1 and Parity 2–4 (Figure 3). As the base DIM increased, the standard deviation of the statistical predictions decreased, whereas that of the ML predictions generally increased. At base DIM 150, the standard deviations of the statistical and ML predictions were 2,282.643 and 1,794.477, respectively, compared with 1,966.770 for the actual cumulative milk yield across all parities. In the parity-specific analyses, the corresponding standard deviations were 1,396.604, 1,229.787, and 1,397.007 for parity 1, and 1,989.416, 1,645.761, and 1,916.177 for parities 2–4. Overall, the statistical predictions more closely reflected the observed between-cow variation, particularly for parity 1, whereas the ML predictions showed consistently lower dispersion than the actual cumulative milk yield. Regarding the prediction horizon, the ML models maintained an overall higher performance for the entire parity, regardless of the base DIM (Figures 4–6). The performance disparity between the two methods was most pronounced when the prediction period was the longest, and the base DIM was the lowest, reaching a maximum MAPE difference of 4.350 at a base DIM of 30 for the 300-day prediction. Conversely, the minimum difference was negligible, 0.024 at base DIM of 150 for the 200-day prediction. Detailed parity analysis further confirmed that, while ML was significantly superior for Parities 2–4 at the early-lactation stages, statistical methods eventually dominated the later stages (base DIM 120 and 150), suggesting that the physiological stabilization of later lactation may favor the structured parameters of statistical models over the flexibility of ML algorithms.

## DISCUSSION

In this study, we developed and evaluated a robust predictive model for long-term milk yield by leveraging daily milking data from early to mid-lactation stages. By using traditional statistical models (SM, AFM, and CCM) with advanced ML algorithms (CatBoost, XGBoost, and RF), we provide a comprehensive comparative analysis of the prediction performance across varying parities and base DIM. Notably, our findings demonstrate the feasibility of shifting from the standard 305-day projection to earlier DIM predictions, offering a practical tool for real-time decision making on commercial dairy farms.

Statistical analysis revealed that the MAPE tended to decrease as the base DIM increased. This trend suggests that as lactation progresses from the early- to mid-stage, the decline in milk yield following peak milk flow is more effectively captured, allowing the individual lactation curve to take a more distinct shape. Furthermore, the accumulation of sufficient cumulative daily milk yield data provides a more robust informational basis, thereby enhancing the predictive precision of the model and reducing relative errors [29]. In particular, the CCM method outperformed SM or AFM in all base DIM, suggesting that the accuracy of total milk yield prediction can be significantly improved when corrections obtained through statistical analysis of SM predictions are applied. Despite utilizing adjustment factors derived from existing detailed large-scale datasets, the AFM showed a slightly lower performance than the CCM because of factors such as the environmental characteristics of the farm, which did not fully match the characteristics of the data used for prediction in this study. These results imply that, if sufficient data have been accumulated, estimating correction factors specific to the dataset may be more effective than directly applying the coefficients derived from previous studies [30].

The difference in MAPE between ML models using all individual daily milk yield data up to the base DIM (e.g., 30 input variables) and those utilizing derived features, such as initial milk yield, short-term cumulative yield, and the calculated slope from the same period, was found to be marginal, at less than 1%. This indicates that condensing 30 days of raw data into key representative variables does not sacrifice predictive accuracy, allowing for a more streamlined model without losing significant information. In a comparative analysis of the developed ML frameworks, CatBoost demonstrated the most robust overall performance, although the margin of superiority among the ML models remained narrow. A notable finding was that at a lower baseline DIM, the ML models consistently outperformed the traditional statistical methods, maintaining lower MAPE values. This underscores the superior capacity of ML to navigate the high volatility and non-linear patterns characteristic of early-stage lactation, where traditional statistical approaches that apply uniform assumptions across individuals may be less effective [31,32]. The contrasting trends in standard deviation should be interpreted relative to the observed variation in actual cumulative milk yield. At base DIM 150, the standard deviations of the statistical predictions were close to the observed values, particularly for parity 1, whereas the ML predictions consistently showed lower dispersion across the overall and parity-specific analyses. Therefore, the increase in the standard deviation of the ML predictions with advancing base DIM does not appear to indicate an overemphasis on late-stage daily fluctuations. Rather, the ML predictions remained relatively concentrated and may have represented between-cow differences in cumulative milk yield more conservatively than the statistical methods. Furthermore, although the ML models generally achieved lower average MAPE values, they occasionally produced substantially larger maximum individual-cow APE values than the statistical models. This finding indicates that favorable average performance did not necessarily ensure stable predictions for every individual cow. The extreme errors may reflect sensitivity to atypical lactation patterns that were insufficiently represented in the training data and therefore suggest a potential risk of local overfitting under the limited sample size [33]. The localized increase in the maximum individual-cow APE at base DIM 120 should not be interpreted as a general decline in predictive accuracy because the mean MAPE continued to decrease over the same period. Instead, this pattern was driven by a limited number of cows with relatively large individual errors and was not consistently observed across all models. Therefore, it was interpreted as individual-level prediction variability rather than a systematic reduction in model performance at mid-lactation.

To characterize milk yield patterns according to parity, the ratio of total milk yield to initial milk yield was calculated, which revealed a distinct divergence in lactation persistence between the 1st parity group and the 2nd to 4th parities. While the ratios for 2nd to 4th parities remained relatively consistent, the 1st parity (primiparous) group exhibited a difference of more than 10% compared with the multiparous groups [34]. This significant variance in production efficiency and yield distribution confirms that primiparous cows follow a fundamentally different lactation curve, necessitating the development of a bifurcated modeling approach, one specifically for the 1st parity and another for subsequent parities, to ensure predictive accuracy. The overall predictive performance across all parities aligned with the averages of the parity 1 (primiparous) and parity 2–4 (multiparous) models. Specifically, in primiparous cows, the mammary gland has not yet reached full physiological development, leading to increased variability in the early lactation curve and a characteristically delayed peak milk yield [19,35,36]. These physiological differences are consistent with those of a previous study demonstrating that primiparous cows possess mammary glands with a lower degree of cellular differentiation but a greater capacity for epithelial cell renewal during lactation [37]. Consequently, mammary epithelial cell proliferation may continue for a longer period after parturition in primiparous cows, leading to a slower increase in milk yield and a delayed peak compared to that in multiparous cows, whose mammary glands are already fully developed at calving. Consequently, SM at a low base DIM tended to produce under- or over-estimations, resulting in higher MAPE values compared with the multiparous group. However, at a higher base DIM, the prediction accuracy for parity 1 surpassed that of multiparous cows because of the higher lactation persistence in primiparous cows, whose slower post-peak decline is more predictable than the rapid decline and high daily fluctuations seen in cows with parity 2 or higher [36]. In the analysis by parity, ML techniques commonly showed superior performance at low DIM; however, when the base DIM increased beyond 120 days, the performance of the statistics-based model increased rapidly, reversing the order of superiority. This suggests that once sufficient data are available to reflect lactation patterns, simpler models may be more suitable for predicting the total milk yield than more complex ML models [38]. Although the ratio analysis supported the separation of parity 1 from parities 2–4, the combined multiparous group should not be interpreted as physiologically homogeneous. Differences in peak yield, lactation persistence, and post-peak decline may remain among parities 2, 3, and 4. Therefore, combining these parities may have increased within-group variance and partially obscured parity-specific prediction patterns, particularly for parity 4. Larger datasets are required to develop and evaluate separate models for each parity.

Another important aspect of this study is that the model performance varies significantly depending on how much total milk yield is predicted up to a certain DIM point. In general, the MAPE increased as the predictive period increased, and the performance degradation was particularly noticeable when predicting up to 300 days at a low base DIM. These results reflect the increased uncertainty associated with estimating the post-peak lactation curve using only the information obtained during early lactation. However, when predicting total milk yield up to 200 days when the base DIM 150 was used, both statistical and ML models showed very low MAPE, which suggests that the mid-lactation period can be a reasonable decision-making point for judging future productivity in terms of field application.

The findings of this study suggest that in dairy farming practice, ML–based models may be more effective during early lactation, whereas simpler and more stable statistical models may be preferable from mid-lactation onwards. We confirmed that developing milk yield prediction models for primiparous and multiparous cows, each with distinct characteristics, is improving prediction accuracy. A total milk yield prediction model for up to 305 DIM can be used to predict farm milk production or establish management strategies for each dairy cow based on economic feasibility. It is also expected that it can be used to monitor the condition of dairy cows by comparing the results of a milk yield prediction model with high prediction accuracy up to earlier DIM points with the actual values. However, the relatively limited size of the dataset employed in this study restricted the development and evaluation of more granular parity-specific models. Although 10-fold cross-validation and a restricted set of predictor variables were used to reduce the risk of overfitting, the ML models were developed and evaluated within the same overall study population. Therefore, their predictive performance may be specific to the farms and production conditions represented in the present dataset, and their generalizability cannot be fully established without external validation. Future studies should evaluate the proposed framework using larger independent datasets encompassing different farms, milking systems, management conditions, and geographic regions. Although the AFM used adjustment factors derived from a large-scale external dataset, its application in the present study was constrained by the absence of information on calving season and exact age in months. Therefore, population-averaged adjustment factors were applied, which may have reduced its ability to account for variation among cows. All statistical and ML models were evaluated using the same information available in the present dataset; however, the AFM may have been more directly affected by the absence of calving season and exact age in months because the original adjustment factors were stratified according to these characteristics. Accordingly, the AFM results should be interpreted as the performance of this approach under limited-information conditions rather than as the maximum predictive performance achievable using the original method. Nevertheless, the present findings suggest that reliable predictive performance may be achieved using routinely collected milk yield records and parity alone. This may support the practical use of the proposed approaches on farms where the information required to apply stratified adjustment factors is not readily available. Because milk production is significantly influenced by health, nutrition, and environmental factors, integrating biological and climatic data into future models will likely enhance early-lactation predictive accuracy [39]. Heat stress during early lactation may reduce initial milk yield, delay the increase toward peak production, and increase short-term daily variation [40]. Consequently, the slope calculated from the early 30-day records may become flatter or more variable and may partly reflect seasonal environmental conditions rather than only individual lactation potential. This effect may also influence the initial average and cumulative milk yield variables. Future inclusion of calving season and temperature–humidity index may improve the interpretation of early-lactation milk yield patterns.

## CONCLUSION

This study developed and evaluated predictive models for estimating long-term cumulative milk yield by leveraging daily production data from the early lactation period to provide a systematic comparison between traditional statistical methods and ML approaches. We demonstrated that, while the predictive accuracy for cumulative milk yield improves as lactation progresses, ML models offer superior performance during the volatile early stage, whereas statistical models provide higher stability from mid-lactation onwards. A key finding is that condensing high-dimensional daily records into key-derived features maintains predictive precision, significantly enhancing the computational efficiency for real-time farm management. Furthermore, the distinct 10% variance in yield patterns between primiparous and multiparous cows underscores the necessity of parity-specific modeling strategies. By adopting a stage-dependent approach that prioritizes ML models during early lactation and statistical models during later stages, the proposed framework demonstrates potential as a decision-support approach for early cattle selection and productivity forecasting. However, its applicability to broader dairy cow populations should be confirmed through external validation using larger and more diverse datasets. The future integration of larger datasets with biological and environmental variables will likely further refine these early-stage predictions.

## Figures and Tables

**Figure 1:**
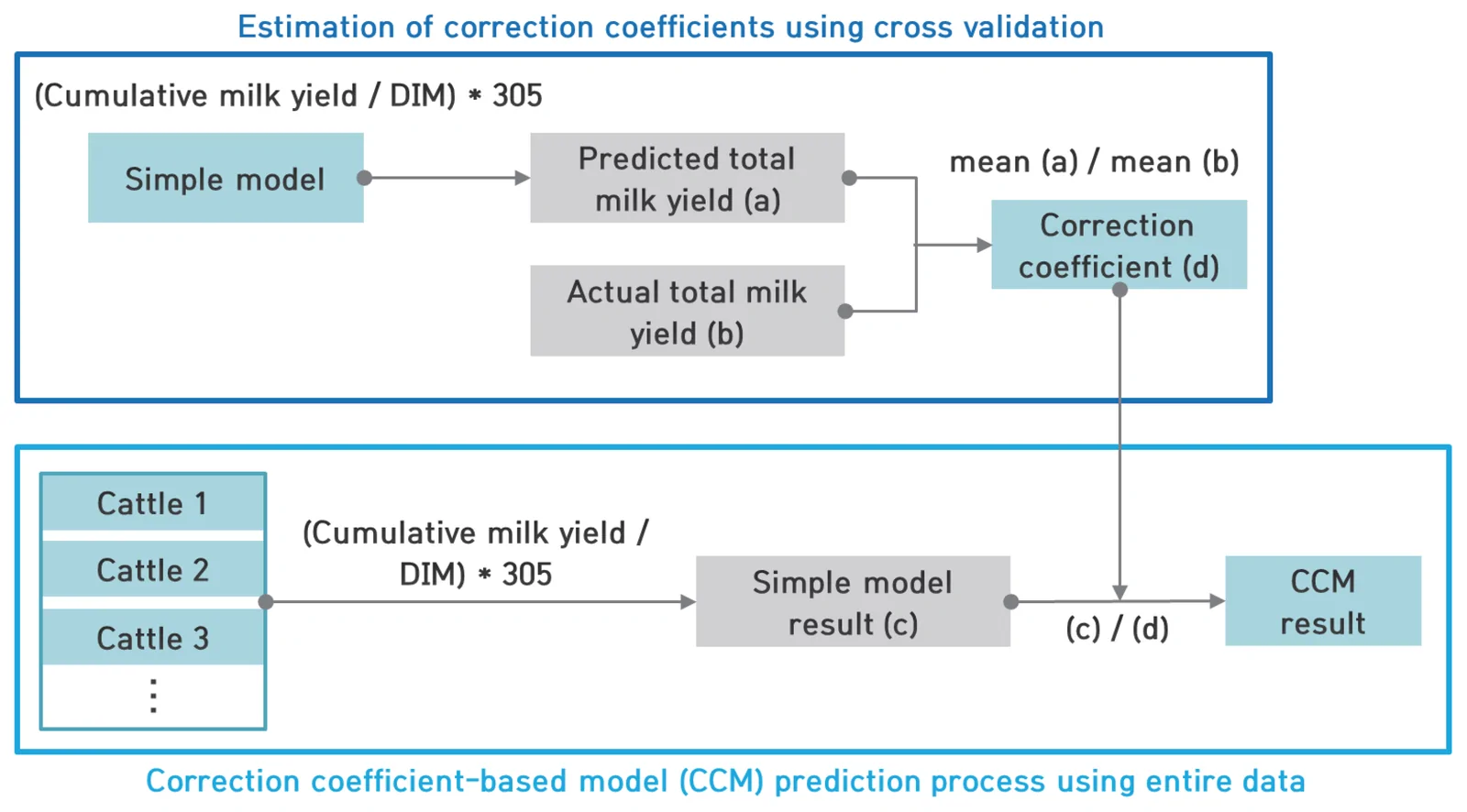
Process of estimating correction coefficients and correction coefficient-based predictions of cumulative milk production. Correction coefficients were estimated using cross-validation by comparing predicted total milk yield with actual total milk yield. The coefficient was calculated as the ratio between the mean predicted value and the mean actual value and was subsequently applied to the predicted values derived from the simple model.

**Figure 2:**
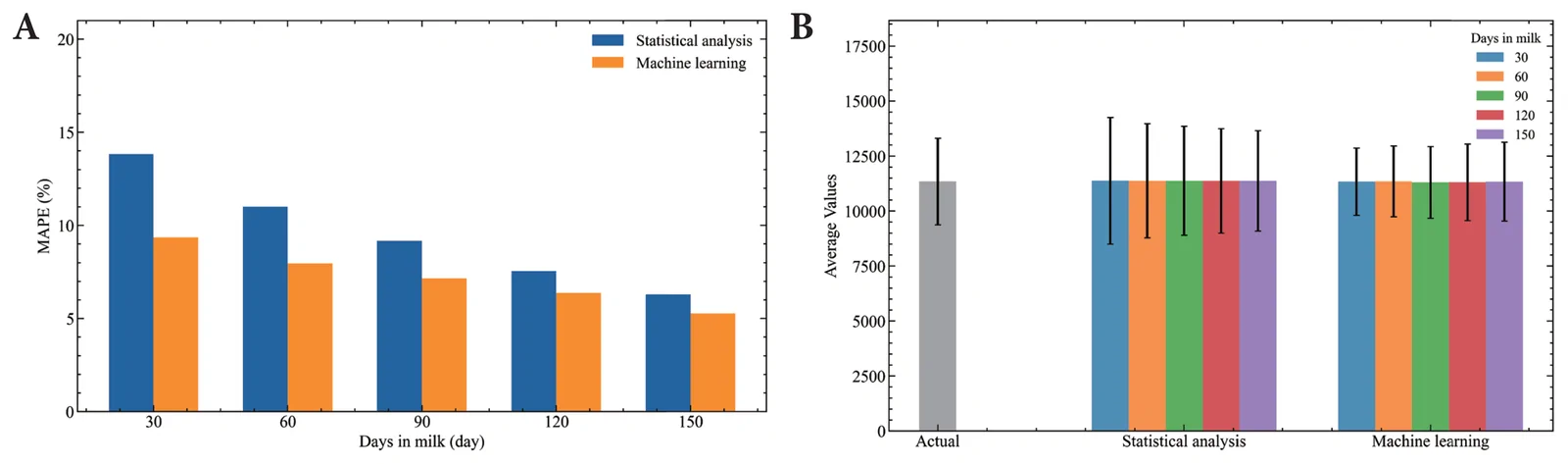
Evaluation of optimized long-term milk yield prediction models developed using statistical analysis and machine learning approaches. (A) Mean absolute percentage error (MAPE) for milk yield predictions at base DIM 30, 60, 90, 120, and 150. (B) Mean actual and predicted milk yields across base DIM periods for statistical analysis and machine learning models. Error bars represent standard deviations. DIM, days in milk.

**Figure 3:**
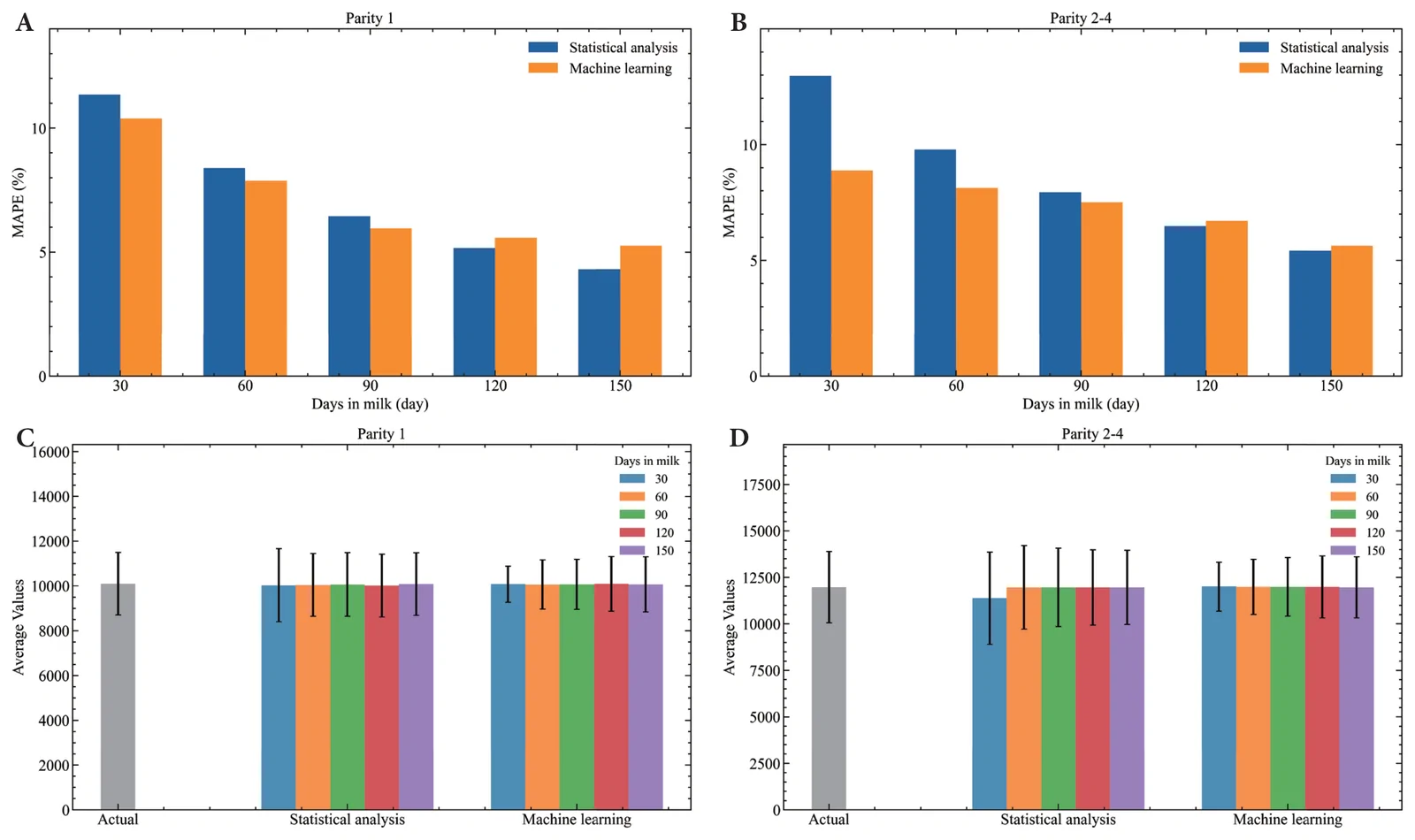
Comparison of optimal long-term milk yield prediction performance between statistical analysis and machine learning approaches according to parity group. (A) Mean absolute percentage error (MAPE) values for long-term milk yield predictions for parity 1 at base DIM 30, 60, 90, 120, and 150. (B) MAPE values for long-term milk yield predictions for parities 2–4 at base DIM 30, 60, 90, 120, and 150. (C) Comparison between actual milk yield and predicted milk yield obtained using statistical analysis and machine learning approaches for parity 1. (D) Comparison between actual milk yield and predicted milk yield for parities 2–4. Error bars represent standard deviations. DIM, days in milk.

**Figure 4:**
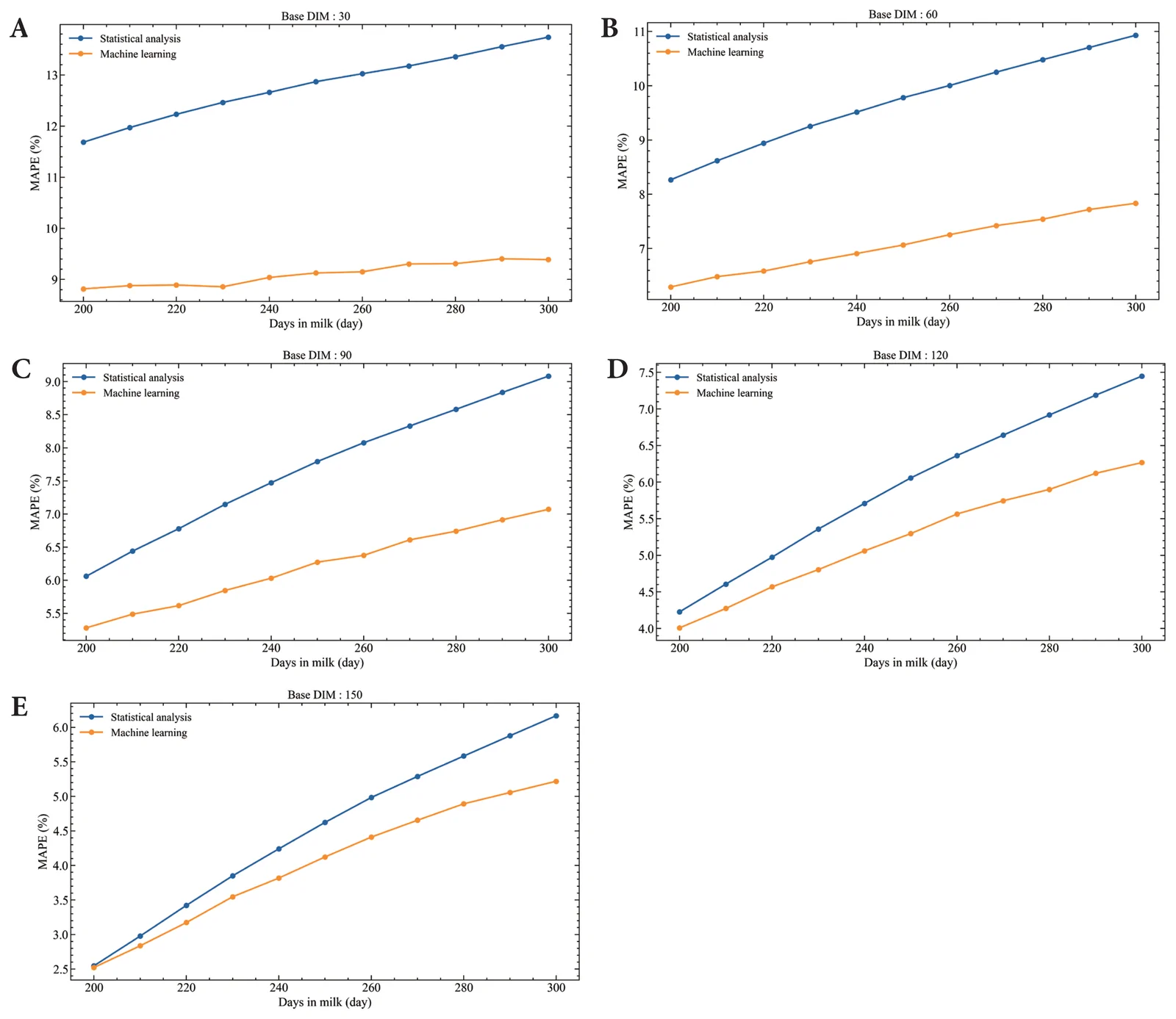
Changes in mean absolute percentage error (MAPE) of statistical analysis and machine learning models across prediction periods from 200 to 300 d in milk (DIM) using different base DIM values. (A) Base DIM 30, (B) base DIM 60, (C) base DIM 90, (D) base DIM 120, and (E) base DIM 150. Lower MAPE values indicate higher prediction accuracy.

**Figure 5:**
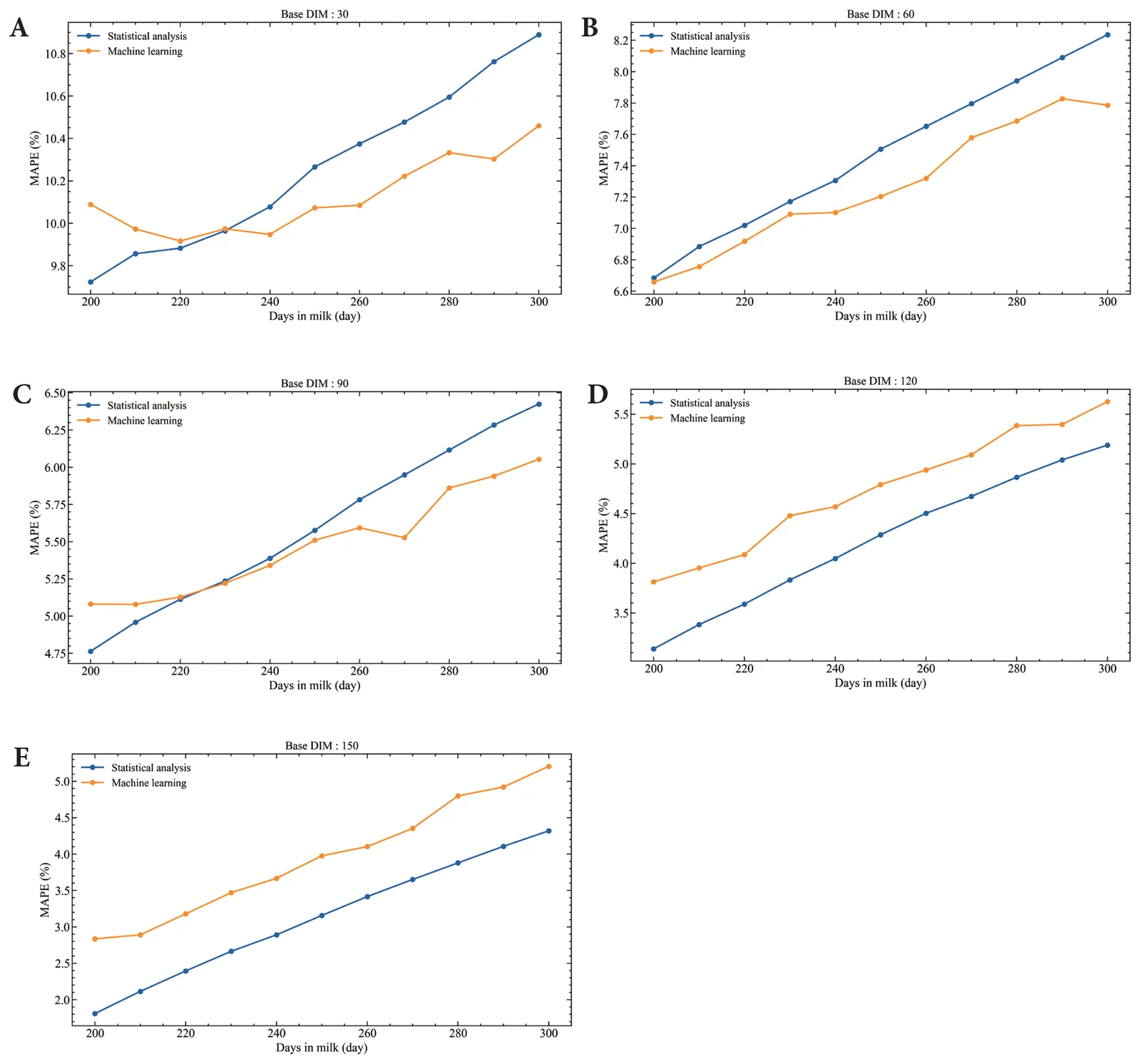
Changes in mean absolute percentage error (MAPE) of statistical analysis and machine learning models across prediction periods from 200 to 300 d in milk (DIM) using different base DIM values for parity 1. (A) Base DIM 30, (B) base DIM 60, (C) base DIM 90, (D) base DIM 120, and (E) base DIM 150. Lower MAPE values indicate higher prediction accuracy.

**Figure 6:**
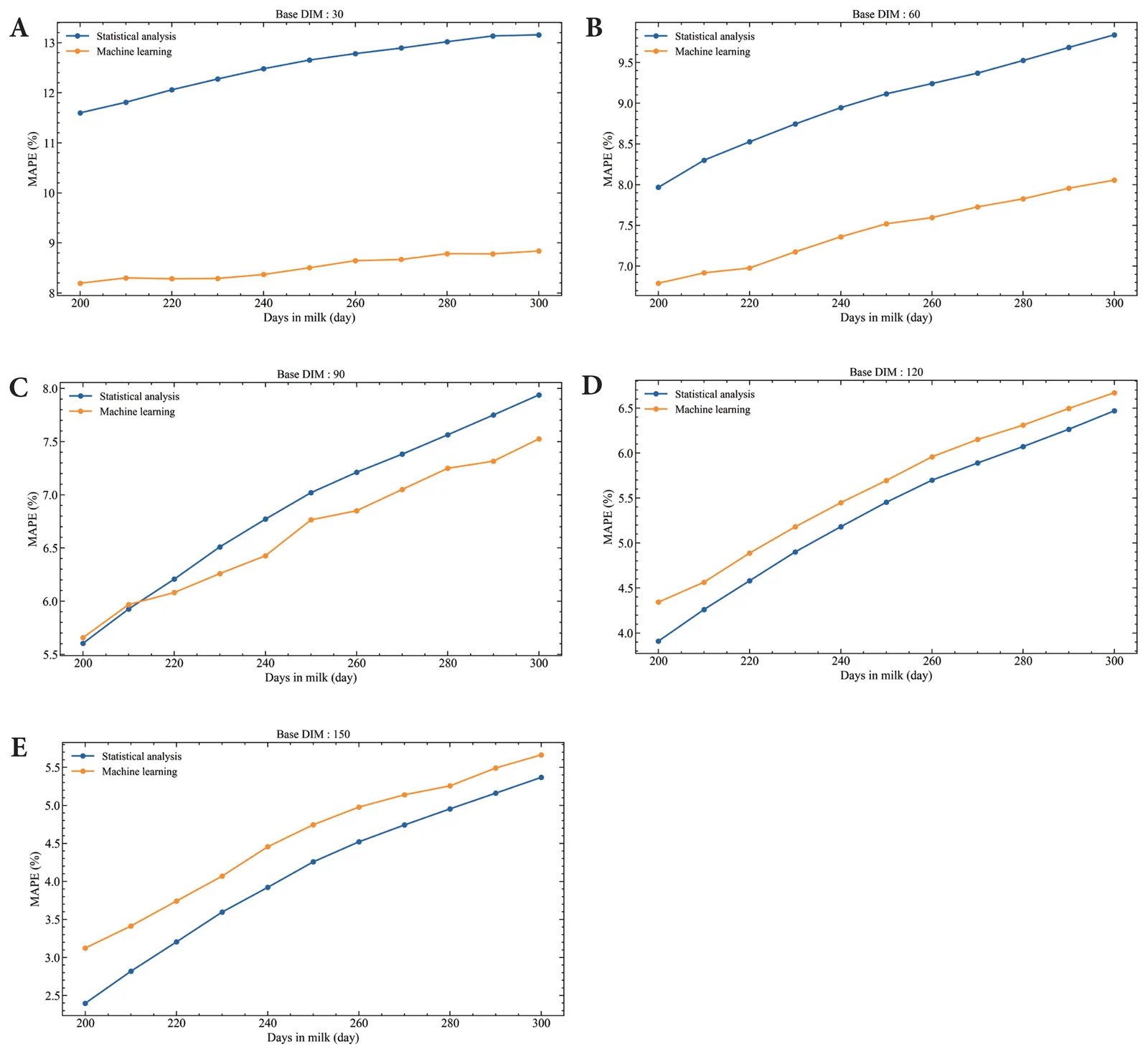
Changes in mean absolute percentage error (MAPE) of statistical analysis and machine learning models across prediction periods from 200 to 300 d in milk (DIM) using different base DIM values for parity 2–4. (A) Base DIM 30, (B) base DIM 60, (C) base DIM 90, (D) base DIM 120, and (E) base DIM 150. Lower MAPE values indicate higher prediction accuracy.

**Table 1:** Mean absolute percentage error of total milk yield prediction by statistical and machine learning models for 305 d

Base DIM	SM (min–max)	AFM (min–max)	CCM (min–max)	CC	CatBoost (min–max)	XGBoost (min–max)	RF (min–max)
30	14.935 (0.083–71.973)	13.896 (0.039–80.310)	13.824 (0.006–89.414)	0.908	9.355 (0.116–61.824)	9.512 (0.018–54.661)	9.438 (0.011–61.177)
60	11.294 (0.112–62.248)	11.040 (0.093–59.205)	10.998 (0.052–58.263)	1.025	7.950 (0.119–45.819)	7.977 (0.002–45.244)	8.223 (0.031–45.978)
90	10.760 (0.110–49.730)	9.351 (0.105–43.505)	9.169 (0.051–40.412)	1.066	7.184 (0.039–35.991)	7.157 (0.003–37.782)	7.330 (0.007–34.716)
120	9.896 (0.134–52.581)	7.843 (0.026–45.277)	7.552 (0.130–42.043)	1.074	6.463 (0.023–39.534)	6.376 (0.020–33.265)	6.530 (0.009–35.791)
150	8.631 (0.025–40.656)	6.504 (0.010–34.061)	6.302 (0.190–31.515)	1.070	5.276 (0.064–27.340)	5.528 (0.002–31.888)	5.864 (0.015–31.392)

DIM, days in milk; SM, simple model; AFM, adjustment factor-based model; CCM, correction coefficient-based model; CC, correction coefficient; RF, Random Forest.

**Table 2:** Mean absolute percentage error of total milk yield prediction by statistical and machine learning models for 305 d by parity

Parity	Base DIM	SM (min–max)	AFM (min–max)	CCM (min–max)	CC	CatBoost (min–max)	XGBoost (min–max)	RF (min–max)
1	30	18.802 (2.518–48.239)	11.348 (0.070–40.020)	11.384 (0.069–40.431)	0.823	10.385 (0.478–51.164)	10.630 (0.034–60.029)	10.883 (0.288–49.488)
	60	9.640 (0.227–30.171)	8.586 (0.439–28.822)	8.392 (0.162–27.732)	0.933	7.909 (0.032–43.229)	7.879 (0.112–39.482)	8.653 (0.399–41.154)
	90	6.529 (0.187–35.427)	7.031 (0.210–32.357)	6.448 (0.007–33.851)	0.976	6.113 (0.072–32.476)	7.167 (0.018–38.522)	7.145 (0.066–41.976)
	120	5.165 (0.134–25.821)	5.798 (0.033–24.152)	5.184 (0.001–25.369)	0.994	5.580 (0.062–31.893)	5.681 (0.064–43.456)	6.146 (0.047–40.012)
	150	4.344 (0.064–19.723)	4.839 (0.057–22.058)	4.311 (0.066–19.285)	1.004	5.261 (0.098–31.824)	5.542 (0.052–37.490)	5.561 (0.131–38.209)
2–4	30	12.974 (0.083–71.973)	15.051 (0.190–61.339)	13.132 (0.201–80.879)	0.951	8.878 (0.083–60.366)	9.172 (0.212–53.252)	8.948 (0.132–59.952)
	60	12.133 (0.112–62.248)	10.164 (0.137–43.023)	9.790 (0.091–51.359)	1.072	8.311 (0.190–47.892)	8.279 (0.010–53.412)	8.121 (0.020–44.666)
	90	12.906 (0.110–49.730)	8.135 (0.057–30.162)	7.946 (0.001–34.637)	1.112	7.737 (0.005–40.047)	7.728 (0.005–47.077)	7.515 (0.005–36.888)
	120	12.296 (0.256–52.581)	6.694 (0.070–33.391)	6.486 (0.127–36.858)	1.115	6.720 (0.027–40.827)	7.157 (0.175–42.881)	6.761 (0.107–34.836)
	150	10.805 (0.025–40.656)	5.668 (0.007–24.722)	5.414 (0.185–27.533)	1.103	5.637 (0.070–32.065)	5.792 (0.051–30.789)	5.766 (0.035–33.125)

DIM, days in milk; SM, simple model; AFM, adjustment factor-based model; CCM, correction coefficient-based model; CC, correction coefficient; RF, Random Forest.

**Table 3:** Comparison of the actual average total milk yield for 305 d with the average results of statistical and machine learning models and the post-hoc test results between the actual and predicted values

Base DIM	SM	AFM	CCM	CatBoost	XGBoost	RF	Average total milk yield
30	10,332.316^*^	10,833.179	11,380.148	11,339.147	11,325.351	11,341.709	11,344.243
60	11,661.408	11,442.709	11,375.027	11,350.424	11,316.196	11,351.396	
90	12,130.694	11,626.375	11,375.823	11,348.141	11,305.909	11,344.119	
120	12,216.509^*^	11,631.719	11,372.789	11,353.184	11,308.099	11,339.426	
150	12,159.419	11,589.322	11,369.224	11,337.677	11,314.733	11,345.000	

* There is a statistically significant difference between the predicted and the actual average total milk yield.

DIM, days in milk; SM, simple model; AFM, adjustment factor-based model; CCM, correction coefficient-based model; RF, Random Forest.

**Table 4:** Comparison of the actual average total milk yield with the average results of statistical and machine learning models by parity and the post-hoc test results between the actual and predicted values

Parity	DIM	SM	AFM	CCM	CatBoost	XGBoost	RF	Average total milk yield
1	30	8,260.627^*^	9,572.306	10,031.518	10,080.376	10,085.529	10,037.783	10,101.233
	60	9,369.246	10,128.002	10,042.288	10,098.037	10,064.877	10,102.455	
	90	9,825.784	10,292.912	10,065.565	10,071.690	10,101.731	10,080.627	
	120	10,016.447	10,317.268	10,077.444	10,090.953	10,079.132	10,088.524	
	150	10,120.436	10,317.867	10,083.474	10,069.327	10,079.170	10,092.556	
2–4	30	11,382.958	10,679.081^*^	11,972.425	11,999.409	11,946.008	12,018.753	11,974.626
	60	12,823.862	11,304.339	11,963.217	11,995.722	11,939.758	11,987.241	
	90	13,299.612^*^	11,561.506	11,959.041	11,973.401	11,956.563	11,992.77	
	120	13,332.255^*^	11,655.451	11,958.414	11,987.730	12,039.387	11,996.599	
	150	13,193.475^*^	11,698.936	11,962.585	11,965.596	11,951.204	11,972.391	

* There is a statistically significant difference between the predicted and the actual average total milk yield.

DIM, days in milk; SM, simple model; AFM, adjustment factor-based model; CCM, correction coefficient-based model; RF, Random Forest.

**Table 5:** MAPE and correction coefficient for statistical and machine learning models for 200 to 300 d with base DIM 30–150

Base DIM	DIM	SM	CCM	CC	CatBoost	XGBoost	RF
30	200	16.631	11.684	0.856	8.817	9.044	9.168
	250	15.689	12.868	0.876	9.128	9.227	9.358
	300	15.158	13.739	0.901	9.389	9.561	9.420
60	200	8.582	8.267	0.967	6.292	6.455	6.925
	250	9.780	9.790	0.990	7.066	7.172	7.422
	300	11.096	10.931	1.019	7.834	7.912	8.112
90	200	6.072	6.060	1.007	5.281	5.906	5.525
	250	8.015	7.792	1.030	6.273	6.824	6.431
	300	10.363	9.082	1.060	7.071	7.789	7.214
120	200	4.308	4.227	1.015	4.119	4.008	4.448
	250	6.741	6.057	1.038	5.420	5.296	5.620
	300	9.512	7.449	1.069	6.391	6.267	6.486
150	200	2.685	2.547	1.012	2.619	2.523	3.115
	250	5.322	4.623	1.035	4.261	4.123	4.719
	300	8.254	6.167	1.065	5.219	5.378	5.787

MAPE, mean absolute percentage error; DIM, days in milk; SM, simple model; CCM, correction coefficient-based model; CC, correction coefficient; RF, Random Forest.

**Table 6:** MAPE and correction coefficient for statistical and machine learning models for 200 to 300 d with base DIM 30–150 by parity

Parity	Base DIM	DIM	SM	CCM	CC	CatBoost	XGBoost	RF
1	30	200	19.582	9.724	0.810	10.118	10.089	10.264
		250	19.444	10.266	0.812	10.073	10.406	10.411
		300	18.949	10.889	0.821	10.460	10.608	10.834
	60	200	9.747	6.684	0.917	6.659	6.766	7.491
		250	9.889	7.506	0.919	7.234	7.204	7.860
		300	9.693	8.236	0.929	7.865	7.786	8.570
	90	200	5.647	4.764	0.960	5.081	5.714	5.450
		250	5.967	5.576	0.962	5.510	6.587	6.068
		300	6.521	6.425	0.973	6.054	7.230	7.030
	120	200	3.509	3.140	0.978	3.814	3.971	4.241
		250	4.348	4.287	0.980	4.792	5.010	5.079
		300	5.189	5.211	0.991	5.627	5.687	6.058
	150	200	2.003	1.810	0.989	2.836	3.000	3.674
		250	3.161	3.157	0.991	3.976	4.362	4.432
		300	4.336	4.319	1.002	5.206	5.837	5.464
2–4	30	200	15.074	11.599	0.880	8.195	8.449	8.392
		250	13.708	12.654	0.909	8.504	8.760	8.640
		300	13.158	13.247	0.944	8.904	9.114	8.839
	60	200	7.967	8.007	0.994	6.790	6.955	6.975
		250	9.723	9.115	1.027	7.669	7.615	7.520
		300	11.836	9.839	1.066	8.320	8.203	8.056
	90	200	6.297	5.604	1.032	5.803	6.007	5.657
		250	9.096	7.020	1.066	6.878	6.786	6.764
		300	12.391	7.939	1.107	7.745	7.617	7.526
	120	200	4.729	3.910	1.035	4.476	4.584	4.343
		250	8.004	5.453	1.069	5.959	6.016	5.695
		300	11.793	6.469	1.110	6.670	7.006	6.701
	150	200	3.045	2.397	1.024	3.218	3.123	3.209
		250	6.463	4.257	1.058	4.751	4.840	4.745
		300	10.322	5.368	1.098	5.703	5.774	5.664

MAPE, mean absolute percentage error; DIM, days in milk; SM, simple model; CCM, correction coefficient-based model; CC, correction coefficient; RF, Random Forest.

## Data Availability

Upon reasonable request, the datasets of this study can be available from the corresponding author.

## SUPPLEMENTARY MATERIAL

**Supplement 1**. Adjustment factors for 305-day milk yield production from a previous study (Cho et al., 2009).

**Supplement 2**. Optimal hyperparameter sets for machine learning algorithms by parity and base DIM.

**Supplement 3**. Mean ratio of cumulative milk yield predicted by the simple model to actual cumulative milk yield according to parity and base DIM.
