# Supplementary material for: A decision support framework for reliable early prediction of long-term cumulative milk yield in dairy cows
Source: Anim Biosci. 2026 Jul 27;39(10):260424. doi: 10.5713/ab.260424 (PMC13662939; doi:10.5713/ab.260424)
Supplement: Supplementary file 1 [file ab-260424-Supplementary-1.pdf]

Supplement 1. Adjustment factors for 305-day milk yield production from a previous study (Cho et al., 2009).

Parity 1

Days in milk	< 30 calving months				≧ 30 calving months				Average
	S1	S2	S3	S4	S1	S2	S3	S4	
30	11.769	11.763	11.817	11.988	11.539	11.611	11.919	11.842	11.781
60	5.489	5.488	5.523	5.598	5.377	5.466	5.464	5.554	5.495
90	3.547	3.54	3.554	3.592	3.523	3.509	3.542	3.592	3.550
120	2.612	2.618	2.625	2.644	2.611	2.598	2.617	2.62	2.618
150	2.066	2.07	2.076	2.09	2.065	2.07	2.075	2.069	2.073

Parity 2-4

Days in milk	< 72 calving months				≧ 72 calving months				Average
	S1	S2	S3	S4	S1	S2	S3	S4	
30	9.217	9.418	9.493	9.432	9.599	9.721	9.722	9.700	9.538
60	4.361	4.448	4.483	4.444	4.487	4.550	4.563	4.514	4.481
90	2.884	2.940	2.967	2.926	2.932	2.980	2.995	2.945	2.946
120	2.184	2.225	2.244	2.205	2.211	2.244	2.255	2.211	2.222
150	1.779	1.809	1.819	1.787	1.792	1.820	1.824	1.790	1.803

[*] S1, S2, S3, S4 mean spring, summer, autumn, and winter, respectively.
