# Supplementary material for: A decision support framework for reliable early prediction of long-term cumulative milk yield in dairy cows
Source: Anim Biosci. 2026 Jul 27;39(10):260424. doi: 10.5713/ab.260424 (PMC13662939; doi:10.5713/ab.260424)
Supplement: Supplementary file 2 [file ab-260424-Supplementary-2.pdf]

Supplement 2. Optimal hyperparameter sets for machine learning algorithms by parity and base DIM.

Parity	Model	Parameter	Base DIM 30	60	90	120	150
Entire	CatBoost	iterations	100	200	200	300	200
		learning_rate	0.08	0.04	0.03	0.04	0.09
		depth	4	3	5	4	3
	XGBoost	n_estimators	100	200	400	100	200
		learning_rate	0.04	0.03	0.01	0.06	0.04
		max_depth	2	2	2	2	2
	Random Forest	n_estimators	300	200	200	200	400
		max_features	2	3	3	5	9
1	CatBoost	iterations	100	100	200	300	200
		learning_rate	0.08	0.09	0.07	0.1	0.08
		depth	4	5	4	4	3
	XGBoost	n_estimators	100	100	200	100	100
		learning_rate	0.01	0.07	0.14	0.06	0.09
		max_depth	2	3	5	3	3
	Random Forest	n_estimators	500	200	200	400	400
		max_features	2	4	7	6	2
2-4	CatBoost	iterations	100	200	100	200	400
		learning_rate	0.07	0.05	0.06	0.12	0.12
		depth	4	5	3	4	5
	XGBoost	n_estimators	100	100	100	500	200
		learning_rate	0.03	0.04	0.03	0.05	0.04
		max_depth	3	2	2	3	2
	Random Forest	n_estimators	100	500	100	300	200
		max_features	3	3	6	8	10

* DIM = days in milk. iterations = the maximum number of trees that can be built; learning_rate = the learning rate is used to reduce the gradient step; depth = depth of the trees; n_estimators = the number of boosting trees (estimators) used in the model; max_features = number of features to consider when finding the best split.
