# Supplementary material for: A decision support framework for reliable early prediction of long-term cumulative milk yield in dairy cows
Source: Anim Biosci. 2026 Jul 27;39(10):260424. doi: 10.5713/ab.260424 (PMC13662939; doi:10.5713/ab.260424)
Supplement: Supplementary file 3 [file ab-260424-Supplementary-3.pdf]

Supplement 3. Mean ratio of cumulative milk yield predicted by the simple model to actual cumulative milk yield according to parity and base DIM.

**Parity**	**Base DIM**				
	**30**	**60**	**90**	**120**	**150**
1	0.818	0.928	0.973	0.992	1.002
2	0.953	1.072	1.110	1.108	1.095
3	0.972	1.085	1.119	1.122	1.111
4	0.905	1.042	1.098	1.110	1.104
